# Évaluation de la couverture thérapeutique de la campagne de traitement de masse contre la filariose lymphatique dans deux districts sanitaires au Burkina Faso

**DOI:** 10.48327/mtsi.v2i4.2022.174

**Published:** 2022-12-13

**Authors:** Mamadou SERME, Adama ZIDA, Roland BOUGMA, Appolinaire KIMA, Christophe NASSA, Micheline OUEDRAOGO, Cathérine KABRE, Harouna ZOROMÉ, Issa GUIRE, Dieudonné NARE, Clarisse BOUGOUMA

**Affiliations:** 1 Programme national de lutte contre les maladies tropicales négligées (PNMTN), Burkina Faso; 2 École doctorale Sciences et Santé, Université Joseph Ki-Zerbo, Ouagadougou, Burkina Faso; 3 Centre National de Formation et de Recherche sur le Paludisme (CNRFP), Ouagadougou, Burkina Faso; 4 Direction régionale de la santé du Centre-Est, Burkina Faso; 5 Helen Keller International, Ouagadougou, Burkina Faso

**Keywords:** Filariose lymphatique, Couverture thérapeutique, Prise supervisée, Traitement de masse, Chimiothérapie préventive, District sanitaire, Tenkodogo, Fada N'Gourma, Burkina Faso, Afrique subsaharienne, Lymphatic filariasis, Therapeutic coverage, Directly observed treatment, Mass treatment, Preventive chemotherapy, Health district, Tenkodogo, Fada N'Gourma, Burkina Faso, Sub-Saharan Africa

## Abstract

**Introduction et justification:**

Afin de valider la couverture thérapeutique rapportée, une enquête indépendante consécutive à la campagne de traitement de masse de médicaments (TDM) a été menée dans les districts sanitaires (DS) de Tenkodogo et Fada N'Gourma en septembre 2018.

**Matériels et méthodes:**

Il s'est agi d'une étude transversale descriptive réalisée dans 30 villages de chaque district du 18 au 24 septembre 2018. La population étudiée était composée de toutes les personnes des communautés visitées. Les variables d’étude comprenaient l’âge, le sexe, l'ingestion des médicaments (ivermectine + albendazole), les événements indésirables et le respect des directives du TDM portant sur la prise supervisée. Les données ont été collectées sur les smartphones via l'application KoBoCollect. Le logiciel Stata Version 14 a été utilisé pour l'analyse des données.

**Résultats:**

Sur les 3 741 personnes interrogées, 53,3% étaient de sexe féminin et l’âge médian était de 14 ans. La couverture épidémiologique enquêtée était de 74% [IC 95% 72-76,1] à Fada N'Gourma et de 79,1% [77,2-80,9] à Tenkodogo, contre des couvertures rapportées de 82,6% et 83% respectivement. Les principales raisons du non-traitement étaient la non-visite de la concession par les distributeurs communautaires (54%) et les absences pendant la distribution de masse de médicaments (43%). Les résultats ont montré que la couverture étudiée était au-dessus du seuil de 65% recommandé par l'OMS, mais inférieure à celle déclarée dans les deux DS. Cependant, des variations importantes de la couverture existent entre les villages. La prise supervisée, ou le traitement directement observé, a été bien respectée (99%).

**Discussion et conclusion:**

Les principaux défis pour améliorer la couverture sont la revisite systématique des ménages ayant enregistré des absents et le ciblage de tous les ménages dans chaque village. Cela devra s'accompagner d'un renforcement de la sensibilisation et de la mobilisation sociale, afin d'amener toutes les personnes éligibles à avaler les médicaments lors des prochaines campagnes.

## Abstract (en)

**Background & rationale:**

Burkina Faso has been implementing preventive chemotherapy against lymphatic filariasis since 2001. While 61 health districts (HDs) have stopped mass drug administration (MDA), transmission persists in 9 HDs despite good reported MDA coverage. To validate the reported coverage, an independent post-MDA survey was conducted in Tenkodogo and Fada N'Gourma HDs in September 2018.

**Materials & methods:**

The study population consisted of all persons in the visited communities. The Coverage survey sample builder (CSSB) tool was used to calculate the sample size and to conduct the random selection of households. A total of 30 villages per HD were selected. The investigators were Ministry of Education agents and health workers not involved in MDA. Data were collected on smartphones through the KoBoCollect application regarding age, sex, drug ingestion (ivermectin + albendazole), adverse events, and whether respondents understood MDA guidelines. Stata Version 14 software was used for data analysis.

**Results:**

A total of 3,741 individuals were surveyed, 53.3% were female and the median age was 14 years. Surveyed epidemiological coverage was 74% [95% CI: 72-76.1] in Fada N'Gourma and 79.1% [95% CI: 77.2-80.9] in Tenkodogo, compared to reported coverages of 82.6% and 83% respectively. Village-level coverage ranged from 32.9% to 100% in Fada N'Gourma and from 56.7% to 93.3% in Tenkodogo. In total, 99% of those treated said they had swallowed the drugs in front of the community drug distributor (CDD) and confirmed the use of dose poles. The main reasons for non-treatment were non-visitation of the compound by CDD (54%) and absences during MDA (43%). Results showed that surveyed coverage was lower than reported coverage in both HDs, yet both were above the 65% threshold recommended by WHO. However, major variations of coverage have been noted among villages. Directly observed treatment appeared to have been well respected.

**Discussion & conclusion:**

The main challenges to increase coverage will be the systematic revisiting of households with absentees and the targeting of all households in each village.

## Introduction

La filariose lymphatique (FL) est l'une des maladies tropicales négligées à transmission vectorielle ciblée pour l’élimination en tant que problème de santé publique par l'Organisation mondiale de la Santé (OMS) [13]. En 2000, plus de 120 millions de personnes étaient infectées, et environ 40 millions d'entre elles souffraient de difformités et étaient handicapées par la maladie [10].

Pour atteindre l’élimination de la FL, l'OMS préconise deux stratégies majeures: i) la chimiothérapie préventive à travers le traitement de masse de médicaments (TDM) aux populations éligibles dans les zones endémiques, et ii) la prise en charge des complications liées à la FL [14].

En 2018, on estimait à 52 le nombre de pays nécessitant une chimiothérapie préventive pour stopper la transmission de cette maladie, afin de protéger 856 millions de personnes qui y vivent [10].

La couverture thérapeutique est considérée comme le principal indicateur de la performance des programmes dans l'administration de masse des médicaments contre la FL [8, 11]. Une couverture thérapeutique efficace (≥ 65%), pendant au moins 4 à 6 campagnes de TDM, est nécessaire pour assurer l'interruption de la transmission de la FL [4]. Il existe plusieurs manières d'estimer la couverture thérapeutique dans le cadre de la chimiothérapie contre la FL. Parmi les types de couvertures, on distingue la couverture thérapeutique rapportée parles distributeurs communautaires (DC) et celle obtenue par une enquête en population [1, 9, 14]. D'une part, la couverture thérapeutique rapportée désigne la proportion de personnes qui ont ingéré la combinaison de médicaments, sur la population totale vivant dans la zone où le TDM a eu lieu. Elle est fournie par les données provenant des rapports des DC. Cette couverture est de peu de valeur si elle n'est pas fiable. Elle est inexacte si l'estimation de la population totale (dénominateur) est erronée, ou si elle comporte des erreurs arithmétiques ou d'agrégation et/ou des falsifications intentionnelles des données dans les registres [8, 11]. C'est pourquoi le programme mondial d’élimination de la FL recommande qu'une enquête de couverture en population soit régulièrement conduite après chaque passage de TDM, afin de valider les couvertures rapportées et de mieux apprécier les performances du programme. D'autre part, la couverture thérapeutique obtenue après enquête en population fournit une estimation plus fiable et ne dépend pas des données agrégées à partir des différents sites de distribution; elle n'est donc pas influencée par les données manquantes, les erreurs de compilation ou les difficultés d'estimation d'un dénominateur exact à partir des données de recensement.

Le Burkina Faso, pays endémique de la FL [4], met en œuvre depuis 2001 une chimiothérapie préventive visant l'interruption de la transmission et l’élimination de la maladie. En 2018, 61 districts sanitaires (DS) sur 70 avaient atteint les critères d'arrêt du traitement de masse. Malgré de bonnes couvertures thérapeutiques rapportées au cours des différentes tournées de TDM, la transmission de la maladie continue dans les 9 autres DS (Batié, Bittou, Bogodogo, Diébougou, Fada N'Gourma, Gaoua, Kampti, Ouargaye et Tenkodogo). En juin 2018, une enquête de pré-évaluation de la transmission de la FL (Pré-TAS) a montré que la proportion de sujets présentant des antigènes filariens circulants (détectés par la bandelette Filariasis Tests Strip, outil diagnostic recommandé par l'OMS [15]) était de 12,5% dans le DS de Tenkodogo et de 9,4% dans celui de Fada N'Gourma, c'est-à-dire bien supérieure au seuil de 2% au-dessous duquel on peut passer à l’étape suivante de suivi-évaluation – l'enquête dévaluation de la transmission proprement dite, en anglais Transmission Assessment Survey (TAS). Cela signifie que le traitement de masse (TDM) n'a pas encore réussi à réduire le niveau de prévalence de l'infection en deçà du seuil d'intervention.

Dans ce contexte de persistance de la transmission de la maladie, le Programme national de lutte contre les maladies tropicales négligées (PNMTN) a identifié, à travers un processus participatif en 2018, des stratégies additionnelles afin de renforcer la qualité de la campagne de distribution de masse contre la FL pour un meilleur impact des interventions. Parmi les stratégies définies figurent le respect de la prise supervisée des médicaments par les DC et la mise en œuvre des enquêtes de couverture post-TDM comme recommandé par l'OMS.

La présente étude a pour but de valider les données de couverture rapportées et d’évaluer l'application de la prise supervisée des médicaments.

## Matériel et Méthodes

### Cadre de l’étude

L’étude s'est déroulée dans les districts sanitaires (DS) de Fada N'Gourma et de Tenkodogo, 2 districts contigus mais situés dans deux régions administratives différentes. Ces districts avaient enregistré des prévalences d'antigénémie filarienne de plus de 50% lors des enquêtes initiales de la cartographie de la FL (52% à Tenkodogo et 56% à Fada N'Gourma). Aussi, ces districts avaient déjà été soumis à au moins 15 années de chimiothérapie contre la FL (16 à Tenkodogo et 15 à Fada N'Gourma) [16].

Le DS de Fada N'Gourma couvre une superficie de 11 200 km^2^ et est l'un des 6 districts sanitaires de la région de l'Est du Burkina Faso. Il couvre les limites administratives de la province du Gourma. La population en 2018 est estimée à 443 115 habitants avec une densité moyenne de 39 habitants/km^2^ [5]. Le district est surtout rural et caractérisé par un profil migratoire important vers les pays voisins comme le Niger, le Bénin, le Togo et la Côte d'Ivoire. L'activité économique est dominée par l'agriculture, l’élevage et les échanges commerciaux, principalement vers le Niger, le Bénin et le Togo.

Son climat est de type soudano-sahélien. Les températures varient de 15 °C à 43 °C et la pluviométrie est irrégulière. Le district est traversé par plusieurs cours d'eau dont les principaux sont la Tapoa et la Singou. De plus, plusieurs retenues d'eau y ont été aménagées dont les barrages de Zanré, Tandjari et Fada N'Gourma.

Sur le plan sanitaire, le paludisme reste la première cause de morbidité et de mortalité. Le traitement de masse contre la FL a commencé en 2003 et au total, 15 tournées annuelles ont déjà été effectuées sans arriver à interrompre la transmission de la maladie. Le nombre de personnes ayant des complications liées à la FL était estimé à 1 463 en 2016, dont 557 porteurs d'hydrocèle.

Le district sanitaire de Tenkodogo, d'une superficie de 1 990 km2, est l'un des 7 districts que compte la région sanitaire du Centre-Est. Situé dans la province du Boulgou, il partage l'un des 4 districts de cette province. Les 3 autres districts sont Zabré, Garango et Bittou. Il couvre une commune urbaine (Tenkodogo avec 6 secteurs et 92 villages).

La population du district sanitaire est estimée à 254 317 habitants en 2018, selon les projections issues du 4e recensement général de la population et de l'habitat (RGPH) de 2006 [5]. Elle est majoritairement jeune et assez pauvre avec 43,9% des habitants vivant en dessous du seuil de pauvreté, estimé à 108 454 Francs CFA/habitant/an. Les mouvements migratoires hors du pays se font principalement vers les pays voisins comme le Ghana et la Côte d'Ivoire, également endémiques à la FL.

Le climat est de type soudano-sahélien caractérisé par l'alternance de deux saisons: une saison sèche de novembre à mai et une saison pluvieuse de juin à octobre.

Le DS de Tenkodogo est traversé par un seul cours d'eau: le Nakambé. Le barrage aménagé de Bagré constitue un important ouvrage hydroélectrique. Il existe d'autres ouvrages hydro-agricoles peu aménagés dans le DS, notamment à Moaga, Ouéguédo et Tenkodogo. Au niveau de ces aménagements hydro-agricoles, on note des comportements assez néfastes pour la santé favorisant le développement des maladies transmissibles vectorielles comme le paludisme et la FL.

L'agriculture est essentiellement basée sur les cultures vivrières (riz, maïs, mil, sorgho) et l’élevage qui constituent les principales activités économiques des populations.

La situation sanitaire à Tenkodogo est également caractérisée par la persistance des endémies locales (paludisme, diarrhées, maladies respiratoires, etc.). Concernant la FL, les TDM sont régulièrement organisés depuis 2002, avec des couvertures thérapeutiques rapportées globalement satisfaisantes. On estime en 2018 à 564 le nombre de personnes porteuses de complications liées à la FL (320 lymphœdèmes et 244 hydrocèles).

**Figure 1:**
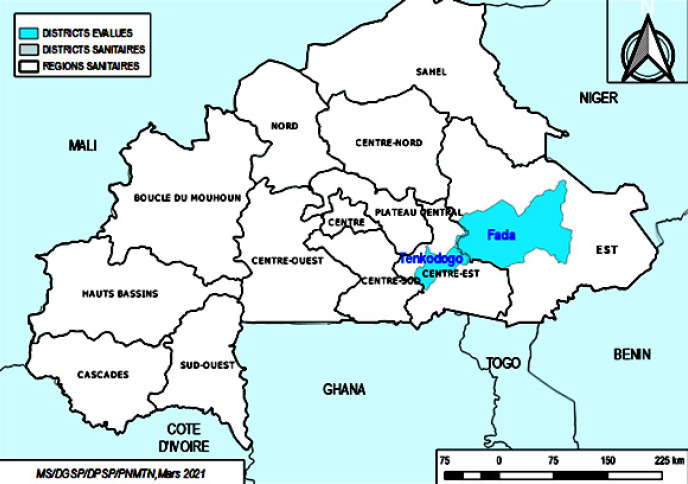
Localisation des districts évalués Location of assessed districts

La Figure 1 présente la localisation des 2 districts évalués.

### Mise en œuvre de la distribution de masse de médicaments

Au Burkina Faso, le TDM contre la FL est assuré en zone rurale par des distributeurs communautaires (DC), et en milieu urbain par des agents de santé accompagnés par des DC qui reçoivent une formation de 2 jours. Les stratégies de distribution utilisées sont le porte-à-porte, couplé avec des postes fixes et le traitement des groupes spécifiques [17]. La combinaison de médicaments utilisée au Burkina Faso est l'ivermectine + albendazole, à cause de la co-endémicité avec l'onchocercose [8, 14] qui interdit l'utilisation de la diéthycarbamazine. Des registres dans lesquels les DC cochent le nombre de personnes traitées, sont utilisés pour le rapportage des données de traitement. Ces registres permettent de rapporter les données par sexe et selon les tranches d’âge suivantes: 5-14 ans et 15 ans et plus. Une toise est utilisée pour déterminer le nombre de comprimés d'ivermectine à administrer à chaque individu. Le nombre de comprimés administrés est d'un comprimé d'albendazole par personne et varie de 1 à 4 comprimés d'ivermectine en fonction de la taille.

### Type d’étude

Nous avons réalisé une enquête transversale descriptive dans 30 villages de chaque district du 18 au 24 septembre 2018, 1 mois après le traitement de masse, afin de respecter les directives de l'OMS qui préconisent que l'enquête de couverture soit effectuée dans les 6 mois suivant la distribution pour éviter des biais de mémoire [14].

### Population d’étude

La population d’étude était composée de toutes les personnes, éligibles ou non au TDM contre la FL, quel que soit leur âge, présentes ou absentes le jour de l'enquête, mais qui résidaient dans les ménages durant la période du traitement de masse (9 au 14 août 2018).

### Échantillonnage

Nous avons utilisé le générateur d’échantillons des enquêtes de couverture pour les maladies tropicales négligées ou *Coverage survey sample builder* (CSSB) pour le calcul de la taille de l’échantillon et la sélection aléatoire des ménages comme recommandé par l'OMS [6]. La taille de l’échantillon nécessaire est calculée selon la formule suivante:

DEFF = design effect = effet de conception, estimé à 4
Z = écart-type associé à un niveau de confiance de 95% = 1,96
p = couverture épidémiologique attendue = 65%
r = taux de non-réponse, estimé à 10%
δ = précision souhaitée = (+/-) 5%

La couverture attendue est le pourcentage de la population supposée avoir ingéré le médicament. Lorsque la couverture attendue n'est pas connue, l'OMS suggère de considérer qu'elle est de 50%. Selon les directives de l'OMS [9] et afin de garantir que la taille de l’échantillon soit suffisante pour atteindre les objectifs de l’étude, il est recommandé de soustraire au moins 15 points du pourcentage de la couverture déclarée. Nous avons considéré une couverture attendue de 65% pour cette enquête, du fait que la couverture rapportée était d'au moins 80% pour les 2 districts. Pour les autres paramètres de calcul de la taille de l’échantillon, nous avons retenu les valeurs par défaut proposées par l'OMS. La taille de l’échantillon générée par l'outil CSSB était estimée à 1 646 personnes par district.

### Taille et nombre de segments

Pour rationaliser l'effort des équipes sur le terrain, chaque village a été divisé en différents « segments ». Chaque segment devait comprendre environ 50 ménages et être enquêté par une équipe donnée. Le nombre total de ménages a été obtenu en divisant la population totale de chaque village par la taille moyenne de personnes vivant dans un ménage qui est de 6 au Burkina Faso [3]. Dans chaque village, le nombre de segments a été obtenu en divisant le nombre total de ménages du village par la taille moyenne d'un segment qui est de 50 ménages.

### Sélection des grappes, ménages et enquêtés

Dans chaque district sanitaire, une liste de tous les villages avec, pour chacun d'entre eux, le nombre de ménages et le nombre de segments de 50 ménages, a été établie. À partir de cette liste, 30 villages ont été sélectionnés, avec une probabilité proportionnelle à leur taille estimée (PPTE), cette taille étant exprimée par le nombre de segments constituant le village. Au sein de chaque village sélectionné, un segment a été choisi de manière aléatoire. Après avoir listé l'ensemble des ménages compris dans le segment choisi, les ménages à visiter pour l'enquête de couverture ont été déterminés en utilisant une liste aléatoire générée par le CSSB (seulement un ménage sur 4 ou 5 est visité). L'enquête portait sur tous les résidents des ménages sélectionnés.

### Collecte et analyse des données

La collecte des données a été réalisée par des enquêteurs indépendants, composés du personnel du Ministère de l’éducation et d'agents de santé non impliqués dans le TDM. Ils avaient tous bénéficié d'une formation théorique et pratique avant le début de l'enquête. Dans chaque village, l’équipe d'enquêteurs a commencé par rencontrer les responsables du village pour expliquer le but de l’étude et obtenir leur consentement à la réalisation de l'activité. La collecte des données a été faite avec l'application mobile KoBoCollect sur des smartphones sur la base d'un questionnaire standardisé. Les caractéristiques socio-démographiques (âge, sexe), l'ingestion de la combinaison de médicaments (ivermectine + albendazole), la survenue et la nature des effets indésirables et l'appréciation des directives de mise en œuvre des TDM (la mesure de la taille par le distributeur communautaire, la prise supervisée) étaient les variables d’étude. La collecte des données a été réalisée dans chaque district par 5 équipes composées de 2 enquêteurs chacune.

Les données collectées ont été exportées du serveur KoBoCollect au format Excel et l'analyse des données a été faite à l'aide du logiciel Stata Version 14.

### Interprétation des résultats

La couverture obtenue lors de l'enquête, ou couverture observée, est appelée couverture épidémiologique. C'est la proportion des personnes enquêtées qui ont avalé la combinaison de médicaments sur le nombre total d'individus enquêtés.

Cette valeur a été comparée au seuil de couverture recommandé par l'OMS qui est de 65% et à la couverture rapportée par les distributeurs communautaires [14] dans chaque district.

### Considérations éthiques

Cette étude a été approuvée par le Ministère de la santé à travers une correspondance administrative de son secrétaire général (n° 2018_2727/MS/SG/DGSP/DPSP/PNMTN). Un consentement éclairé a été obtenu chez tous les enquêtés. Les parents ont souvent répondu pour les jeunes enfants (moins de 5 ans) ou les absents. Le traitement par l'ivermectine et l'albendazole était administré gratuitement aux personnes qui n'avaient pas été traitées durant la campagne.

## Resultats

### Caractéristiques des personnes enquêtées

L'enquête a été réalisée dans 30 villages dans chaque district comme prévu. Au total, 606 ménages ont été visités dont 305 à Tenkodogo et 301 à Fada N'Gourma.

Au total, 3 741 individus ont été enquêtés (1 934 à Fada N'Gourma et 1 807 à Tenkodogo). L’échantillon enquêté était constitué de 15,8% d'enfants de moins de 5 ans, 35,4% d'enfants de 5-14 ans et 48,8% d'adultes de 15 ans et plus. L’âge moyen était de 20,4 ans avec une médiane de 14 ans et 53,3% des enquêtés étaient de sexe féminin. Le nombre moyen de personnes enquêtées par ménage était de 6,2 dans les 2 districts. Les caractéristiques socio-démographiques des enquêtés sont présentées dans le Tableau I.

**Tableau I:** Caractéristiques socio-démographiques des personnes enquêtées lors de l’évaluation de la couverture thérapeutique du traitement de masse contre la filariose lymphatique dans les districts sanitaires de Tenkodogo et Fada N'Gourma en 2018 Sociodemographic characteristics of the respondents during the coverage survey of lymphatic filariasis mass drug administration in the health districts of Tenkodogo and Fada N'Gourma in 2018

Caractéristiques	Effectif	Pourcentage (%)
**Ensemble (n = 3741)**		
District		
Fada N'Gourma	1 934	51,5
Tenkodogo	1 807	48,3
Sexe		
féminin	1 994	53,3
masculin	1 747	46,7
Âge (en années)		
moins de 5 ans	592	15,8
5-14 ans	1 323	35,4
15 ans et +	1 826	48,8
**District de Fada N'Gourma (n = 1934)**		
Sexe		
féminin	1 002	51,8
masculin	932	48,2
Âge (en années)		
moins de 5 ans	309	16,0
5-14 ans	696	36,0
15 ans et +	929	48,0
**District de Tenkodogo (n = 1807)**		
Sexe		
féminin	992	54,9
masculin	815	45,1
Âge (en années)		
moins de 5 ans	283	15,7
5-14 ans	627	34,7
15 ans et +	897	49,6

### Couverture thérapeutique

Dans le district sanitaire de Fada N'Gourma, sur 1 934 personnes enquêtées, 1 432 ont déclaré avoir ingéré les médicaments, soit une couverture épidémiologique de 74% avec un intervalle de confiance à 95% de [72-76,1] pour une couverture épidémiologique rapportée de 82,6%.

À Tenkodogo, avec 1 807 personnes enquêtées, la couverture était de 79,1% avec un intervalle de confiance de 95% de [77,2-80,9] (1 430 ont déclaré avoir avalé les médicaments) contre une couverture épidémiologique rapportée de 83% (Tableau II).

**Tableau II:** Couverture épidémiologique des personnes enquêtées selon leurs caractéristiques Epidemiological coverage surveyed by respondent characteristics

Caractéristiques	Avalé IVM+ALB	Pourcentage (%)	IC 95%
District			
Fada	1 432	74,0	[72-76,1]
Tenkodogo	1 430	79,1	[77,2-80,9]
Sexe			
féminin	1 542	77,8	[76,0-79,6]
masculin	1 310	75,0	[72,9-77,0]
Âge			
5-14 ans	1 212	91,6	[90,1-93,1]
15 ans plus	1 650	90,4	[88,9-91,6]
Ensemble	2 862	76,5	[75,1-77,8]

IVM: ivermectine

ALB: albendazole

Il n'existe pas de différence significative entre la couverture thérapeutique selon les groupes d’âge ciblés (Chi^2^ = 1,44; p = 0,23) par le traitement de masse. Par contre, nos résultats montrent que la couverture chez les sujets de sexe masculin est inférieure à celle chez les sujets de sexe féminin (Chi^2^ = 4,2; p = 0,04). La couverture dans le district de Tenkodogo est supérieure à celle enregistrée à Fada N'Gourma (Chi^2^ = 13,48; p < 0,0001). La différence statistique entre la couverture rapportée et la couverture épidémiologique pour chaque localité est significative (p < 0,0001). La variation entre la couverture lors de l'enquête et celle rapportée est de -8,6 points à Fada N'Gourma et -3,9 points à Tenkodogo (Tableau III).

**Tableau III:** Comparaison des couvertures rapportées et épidémiologiques du traitement de masse contre la filariose lymphatique en 2018 dans les deux districts sanitaires Comparison of reported and epidemiological coverage of lymphatic filariasis mass drug administration in 2018 in the two health districts

Districts	Couverture rapportée (%)	Couverture épidémiologique (%)	Validation de la couverture rapportée	Variation (%)
Fada	82,6	74,0 [72-76,1]	Non	-8,6
Tenkodogo	83,0	79,1 [77,2-80,9]	Non	-3,9

### Raisons de non-traitement

Au total, 210 personnes éligibles n'avaient pas été traitées. Les principales raisons du non-traitement chez les personnes éligibles étaient les absences (43%), la non-visite du ménage par le DC (54%), le refus (2%) et la rupture de stock de médicaments (1%).

### Application des directives

Concernant l'appréciation du respect des directives, 99% des personnes ayant reçu les médicaments ont déclaré avoir avalé les médicaments devant les DC. Une même proportion a confirmé l'utilisation effective de la toise par le DC, avant l'offre des médicaments.

### Effets indésirables

Au total, 98 (0,3%) personnes ayant avalé les médicaments ont déclaré avoir développé des effets indésirables, avec respectivement 26 à Fada N'Gourma et 72 à Tenkodogo. Les difficultés respiratoires, la somnolence, la diarrhée et les nausées étaient les effets indésirables évoqués le plus fréquemment par les enquêtés (Tableau 4). On note que 1,5% des personnes ayant avalé les médicaments ont développé des troubles digestifs (diarrhée, nausée, maux de ventre).

**Tableau IV:** Fréquence des effets indésirables mineurs chez les personnes ayant avalé les médicaments (n = 2 862) Frequency of minor side effects in people who have swallowed the medicines (n=2862)

Effets	Effectif	Pourcentage (%)
Somnolence	34	1,19
Diarrhée	15	0,52
Nausée	15	0,52
Vertiges	14	0,49
Maux de ventre	14	0,49
Maux de tête	12	0,42
Nodules /gonflement	3	0,10
Éruption cutanée	2	0,07
Difficultés respiratoires	3	0,10
Autres	16	0,56

## Discussion

La chimio-prévention est l'une des principales stratégies d'intervention utilisées par les programmes de contrôle et d’élimination de 5 maladies tropicales négligées (MTN) dont la FL [7].

L’évaluation de la couverture thérapeutique est un outil précieux pour mesurer la performance d'un programme d’élimination de la FL. Plusieurs méthodes d’évaluation de la couverture thérapeutique post-TDM ont largement été utilisées dans le passé sur le terrain: la méthode statistique d’échantillonnage par lot ou Lot Quality Assurance Sampling (LQAS), le sondage en grappes à 2 degrés (méthode du Programme élargi de vaccination ou PEV) [1]. Il est cependant reconnu depuis longtemps que l'approche du PEV risque de fournir des résultats biaisés, car elle n'utilise pas un échantillonnage aléatoire dans lequel chaque membre de la population a une chance égale d’être sélectionné. Quant à l'approche LQAS, qui est reconnue pour la petite taille de l’échantillon, elle d'offre pas une meilleure représentativité de l'indicateur [9].

En 2016, tenant compte du potentiel de biais de ces deux méthodes, le groupe de revue des programmes MTN de l'OMS recommande l’évaluation de la couverture thérapeutique dans le cadre de ces programmes selon l'approche d’échantillonnage aléatoire avec segmentation, en anglais Probability Sampling with Segmentation (PSS) qui offre un échantillonnage de probabilité égale [12]. Cette dernière méthodologie approuvée par l'OMS a été utilisée pour ces deux enquêtes, qui ont été réalisées environ un mois après le TDM.

L’échantillon requis a été atteint à la fin de l'enquête dans les deux districts. La couverture épidémiologique observée dans les deux DS est supérieure au seuil de couverture attendue recommandé par l'OMS (≥ 65%) [5].

La couverture thérapeutique observée sur le terrain dans les deux districts semble inférieure à la moyenne rapportée par les distributeurs. Ces résultats corroborent ceux trouvés par d'autres auteurs [21] qui ont montré que 73,3% des couvertures rapportées étaient surestimées. Toutefois, la variation entre la couverture rapportée et celle observée s'inscrit dans la fourchette des 15% de différence de l'OMS [9, 12].

Bien que, globalement, les couvertures observées dépassent le seuil attendu, l'analyse au niveau des villages montre quelques disparités souvent importantes. Les résultats de l’étude ont montré que 18 villages avaient des couvertures inférieures à 75% dont 10 avec des couvertures inférieures à 65%, ce qui, selon les directives nationales, devrait déclencher une action de rattrapage.

La faible couverture dans certains villages pourrait s'expliquer par l'insuffisance de démarcation géographique des zones à traiter par chaque DC, ou par un trop grand nombre de ménages affectés à la distribution des médicaments par les DC [19].

La nécessité de renforcer la mobilisation sociale et la sensibilisation dans les zones à faible couverture thérapeutique aux prochains TDM et de revisiter systématiquement les ménages ayant enregistré des absents s'impose, afin d'accroître la couverture et de la maintenir à des niveaux suffisants dans tous les villages. En effet, l’étude a montré que les absences pendant le passage des équipes de DC restent la principale raison de non-traitement.

Des efforts devront être fournis par le programme, afin de suivre les couvertures thérapeutiques au niveau de chaque village et de documenter les raisons des contre-performances. Cela permettra de proposer des stratégies adaptées, afin de résoudre tout problème de faible couverture qui est un frein à la réduction de la prévalence en dessous du seuil d'intervention [2].

Dans les 2 districts, les couvertures rapportées se situent en dehors de l'intervalle de confiance à 95% des couvertures observées après l'enquête, c'est-à-dire non validées [14], traduisant un problème de qualité du système de collecte des données. Dans ces 2 districts, aucune évaluation de la qualité n'a été faite auparavant. La mise en œuvre d'une évaluation de la qualité des données (DQA) permettra d'identifier les insuffisances du système de collecte des données et de proposer des actions correctrices. Aussi, le renforcement du suivi de la qualité des données pendant le TDM à travers le suivi journalier des données et le contrôle de qualité pendant la supervision seront nécessaires.

L’étude a montré que les directives de mise en œuvre des TDM se rapportant au traitement directement observé et à la prise de la taille avant d'administrer le médicament semblent avoir été bien respectées. Le traitement directement observé est une forte recommandation de l'OMS, car il permet de s'assurer de l'ingestion effective des médicaments par les populations [20]. L'application de ces directives, combinée au maintien d'une couverture suffisante dans tous les villages, pourrait permettre d'obtenir un meilleur résultat aux prochaines évaluations épidémiologiques.

Toutefois cette étude n'a pas permis d’évaluer l'assiduité individuelle au traitement de masse pendant plusieurs campagnes, du fait que les supports de collecte des données n’étaient pas nominatifs [18].

## Conclusion

Les résultats de cette enquête montrent des couvertures épidémiologiques inférieures aux couvertures rapportées dans les 2 districts, mais qui sont globalement satisfaisantes par rapport à la valeur attendue de 65%. L'application effective de la prise supervisée des médicaments semble avoir été respectée. Les principaux défis à relever pour accroître davantage la couverture thérapeutique sont la revisite systématique des ménages ayant enregistré des absents et le ciblage de tous les ménages de chaque village. L'application de la prise supervisée, combinée à une assiduité au traitement au niveau individuel pendant plusieurs années, pourrait permettre d'obtenir le résultat souhaité dans les années à venir. De futures recherches sont nécessaires pour évaluer, d'une part l'assiduité continue au traitement de masse au niveau individuel, et d'autre part l'importance des personnes jamais traitées au sein des communautés ainsi que la conduite des évaluations de la qualité des données transmises.

## Contributions Des Auteurs

Auteur principal, rédaction, analyse des données: Mamadou SERME

Lecture et analyse des données: Christophe NASSA, Harouna ZOROMÉ

Lecture et correction: Adama ZIDA, Roland BOUGMA, Appolinaire KIMA, Cathérine KABRE, Issa GUIRE, Clarisse BOUGOUMA, Micheline OUEDRAOGO Résumé anglais: Dieudonné NARE

## Remerciements

La réalisation de cette étude est le fruit d'efforts conjugués des différents acteurs impliqués. Le Programme national de lutte contre les maladies tropicales négligées remercie la Banque mondiale, à travers le Programme d'appui au développement sanitaire (PADS) pour le financement de cette activité. Les remerciements vont également aux autorités sanitaires, aux enquêteurs, aux superviseurs, aux personnes enquêtées et aux populations des villages visités. Enfin les remerciements s'adressent à l’équipe de Helen Keller International pour la traduction du résumé en anglais.

## Liens D'intérêts

Les auteurs déclarent ne pas avoir de liens d'intérêts.
